# Cancer CRC: A Comprehensive Cancer Core Transcriptional Regulatory Circuit Resource and Analysis Platform

**DOI:** 10.3389/fonc.2021.761700

**Published:** 2021-10-12

**Authors:** Ling Wei, Jiaxin Chen, Chao Song, Yuexin Zhang, Yimeng Zhang, Mingcong Xu, Chenchen Feng, Yu Gao, Fengcui Qian, Qiuyu Wang, Desi Shang, Xinyuan Zhou, Jiang Zhu, Xiaopeng Wang, Yijie Jia, Jiaqi Liu, Yanbing Zhu, Chunquan Li

**Affiliations:** ^1^ School of Medical Informatics, Daqing Campus, Harbin Medical University, Daqing, China; ^2^ The First Affiliated Hospital, Institute of Cardiovascular Disease, Hengyang Medical School, University of South China, Hengyang, China; ^3^ Cardiovascular Lab of Big Data and Imaging Artificial Intelligence, The First Affiliated Hospital, Hengyang Medical School, University of South China, Hengyang, China; ^4^ School of Computer, University of South China, Hengyang, China; ^5^ Hunan Provincial Base for Scientific and Technological Innovation Cooperation, University of South China, Hengyang, China; ^6^ Experimental and Translational Research Center, Beijing Friendship Hospital, Capital Medical University, Beijing, China; ^7^ Beijing Clinical Research Institute, Beijing, China; ^8^ General Surgery Department, Beijing Friendship Hospital, Capital Medical University, Beijing, China; ^9^ Guangxi Key Laboratory of Diabetic Systems Medicine, Guilin Medical University, Guilin, China

**Keywords:** cancer, core transcriptional regulatory circuit (CRC), transcription factors (TFs), super-enhancers (SEs), chromatin accessibility

## Abstract

A core transcriptional regulatory circuit (CRC) is a group of interconnected auto-regulating transcription factors (TFs) that form loops and can be identified by super-enhancers (SEs). Studies have indicated that CRCs play an important role in defining cellular identity and determining cellular fate. Additionally, core TFs in CRCs are regulators of cell-type-specific transcriptional regulation. However, a global view of CRC properties across various cancer types has not been generated. Thus, we integrated paired cancer ATAC-seq and H3K27ac ChIP-seq data for specific cell lines to develop the Cancer CRC (http://bio.liclab.net/Cancer_crc/index.html). This platform documented 94,108 cancer CRCs, including 325 core TFs. The cancer CRC also provided the “SE active core TFs analysis” and “TF enrichment analysis” tools to identify potentially key TFs in cancer. In addition, we performed a comprehensive analysis of core TFs in various cancer types to reveal conserved and cancer-specific TFs.

## Introduction

Transcription factors (TFs) regulate gene expression by binding to specific DNA sequences (1). A small number of TFs that are expressed in each cell type control the gene expression programs in specific cells, and dysregulation of these genes can cause various diseases (2–6). These TF clusters play crucial roles in the pathology and development of cancers. For example, in *MYCN*-amplified neuroblastoma, the TFs *MYCN*, *HAND2, ISL1, PHOX2B, GATA3*, and *TBX2* are essential for maintaining cell state and survival (7). Ran *et al.* reported that in gastrointestinal stromal tumors (GIST), the forkhead family member *FOXF1* directly controls the transcription of two master regulators, *KIT* and *ETV1*, and these TFs are required for precursor-interstitial cell lineage specification and the tumorigenesis of GIST (8). Furthermore, groundbreaking studies on embryonic stem cells (ESCs) revealed that *OCT4, SOX2*, and *NANOG* played prominent roles in early cell development. These TFs are essential for the proliferation of undifferentiated ESCs and contribute to the pluripotency and self-renewal of stem cells (9–12). Together, these TFs cooperate to regulate cellular gene expression by binding regulatory elements that impact expressed genes. Additionally, they not only bind to their own loci, but also regulate mutually. They collaborate to form regulatory circuitry and their expression is driven individually and in cooperation with other members of the circuit. Researchers have defined these interconnected auto-regulatory loops as core transcriptional regulatory circuits (CRCs), and TFs within these CRCs are known as core TFs (9, 13). An increasing number of studies have investigated CRCs and core TFs in different cancer types. These studies have shown that core TFs promoted the growth of cancer cells (14). For example, in esophageal squamous cell carcinoma, the TFs *TP63, SOX2*, and *KLF5* form a CRC to control gene expression programs and transcription patterns (15). Additionally, Decaesteker *et al.* showed that *TBX2* is a member of a CRC in neuroblastoma. Furthermore, *TBX2* drives proliferation through the activation of *FOXM1* target genes (16). Above all, CRCs are critical for maintaining transcriptional patterns. Identification of CRCs in various cancers is essential for a better understanding of cell/tissue characteristics and for addressing fundamental molecular and cellular biological questions.

Studies have shown that genes encoding TFs that form CRCs are driven by SEs (17). SEs are densely bound by an array of TFs (18, 19). Additionally, SEs are widely thought to drive the expression of cell-type-specific or disease-associated genes, including core TFs (5, 17, 20–22). Recently, a CRC database, dbCoRC, was developed by Hung *et al.* (23). This database provides a valuable resource to catalog the fast-expanding information on CRCs. In dbCoRC, CRCs are computationally inferred from the mapping of SEs and the prediction of TF binding sites using H3K27ac ChIP-seq data. However, chromatin accessible regions can now be identified by the “assay for transposase-accessible chromatin using sequencing” (ATAC-seq) (24) technology. For H3k27ac ChIP-seq and ATAC-seq data, both can be used independently to identify regulatory element regions. Uniquely, the combination of these two kinds of data can be used to identify precise TF-binding hypersensitive elements within large enhancer regions, which will be helpful for identifying SE-defined TF networks in cancer. Furthermore, the python package “Coltron” (https://pypi.python.org/pypi/coltron) developed by Saint-Andre *et al.* can be used to integrate H3K27ac ChIP-seq and ATAC-seq data to identify CRCs (17, 25). For instance, Ott *et al.* used “Coltron” to generate regulatory landscapes of chronic lymphocytic leukemia samples and representative cell lines. They showed that CRC discovery in primary cancer cells uncovered tumor-specific hallmarks and active TF regulatory pathways (26). That study also demonstrated the success of CRC reconstruction using “Coltron”.

Here, we present the Cancer CRC (http://bio.liclab.net/Cancer_crc/index.html), the first comprehensive and interactive resource of cancer CRC models. To highlight the advantages and innovations of Cancer CRC, we compared it with existing CRC databases dbCoRC in both data and functionality in **Table S1**. Cancer CRC focuses on building a comprehensive cancer CRCs and analysis platform, and has more obvious cancer specificity and functional advantages (**Table S1**). Cancer CRC contains information on core TFs, including topological properties (in-degree and out-degree), expression values, frequencies, associated SEs, mutational information, disease information, associated pathways, Gene Ontology (GO) functional annotations and survival analyses. In particular, Cancer CRC supports a TF enrichment analysis tool to enable the discovery of important cancer-related TFs. Here, we provide a case study using differentially expressed TFs in breast cancer to demonstrate the value of this analytical tool. Moreover, we describe a global analysis of all core TFs and reveal the universality and specificity of TFs across cancer types. The Cancer CRC platform is a valuable resource to query, browse and visualize information about cancer CRCs, and it can also be used to investigate the function of TFs in cancer types.

## Materials and Methods

### Data Sources and Processing

#### Super Enhancers

First, we collected raw H3K27ac ChIP-seq data from NCBI GEO/SRA, ENCODE, Roadmap and the Genomics of Gene Regulation Project. In the process of data collection and collation, it’s required that H3K27ac and the corresponding control group (Input) ChIP-seq data must be both present in a cell or tissue sample to reduce false positives. Second, we used Bowtie (27) to align the raw sequencing reads to the human (hg19) reference genome. Third, the ChIP-seq peak calling analysis was performed using MACS software (28). Last, we used rank ordering of super enhancers (22) software to annotate SE regions. Furthermore, detailed SE information and analysis can be viewed in the SEdb (29) database and SE analysis (30) web server, which were developed by our group.

#### Accessible Chromatin

ATAC-seq provides a systematic approach to understand the genome in cancer to advance diagnosis and therapy. To obtain cancer-specific open chromatin regions, we collected genomic regions of 23 kinds of primary human cancers from TCGA (https://www.cancer.gov/). These genomic regions have gone through quality control and screening, which are high-quality and cancer type-specific peak set. For each sample, the MACS2 callpeak command with the parameters “–shift -75 –extsize 150 –nomodel –call-summits –nolambda –keep-dup all -p 0.01” were used for peak calling. Then, the peak summits were extended by 250 bp on either side to a final width of 501 bp, filtered by the ENCODE hg38 blacklist (https://www.encodeproject.org/annotations/ENCSR636HFF/), and further filtered to remove peaks that extend beyond the ends of chromosomes (31). Then, we used the UCSC liftOver tool (32) to convert the genomic locations of these regions onto the hg19 reference genome to match to the SE data.

### CRC Construction

Based on the available datasets, we only retained paired samples with both H3K27ac ChIP-seq data and cancer ATAC-seq data, which covered 13 cancer tissue types. The detailed information on each H3k27ac sample (source, tissue type, sample type and cancer type) can be found in **Table S2**. To integrate the two types of data for each cancer type, the Coltron pipeline was used with the command: “coltron [options] -e [ENHANCER_FILE] -b [BAM_FILE] -g [GENOME] -o [OUTPUTFOLDER] -n [NAME]”, along with the option “-s SUBPEAKS, –subpeaks=SUBPEAKS”. We also provide the detailed technical pipeline (**Figure S1**).

By calculating the regions overlapping between the H3k27ac ChIP-seq and ATAC-seq data, we obtained the trans-active SEs regions that TFs typically bind to. At present, there are many ways to recognize TF binding motifs (33, 34). Here, the underlying sequences from these regions were extracted and FIMO v4.91 was used to search for TF binding sites in these regions (35). TF position-weight matrices were taken from TRANSFAC and JASPAR (36, 37). When a motif of TF A was identified in an SE of TF B, an edge was linked between TF A and TF B. We merged all edges to obtain a TF interaction network. A clique was defined as a subnetwork of the TF network with a size of at least two nodes (TFs) where all TFs were connected to themselves and other TFs within that clique. For each sample, the set of all cliques of size 2 or greater were defined. Additionally, CRCs were defined as completely auto-regulatory TF cliques within each sample.

Through our pipeline, we obtained the CRC file of each sample. Taking the esophageal cancer KYSE510 cell line as an example (**Table S3**), the results file consisted of three columns (a list of all CRCs in the sample, the score of each CRC and the number of TFs of each CRC). Ultimately, we identified more than 94,000 CRCs, and the distribution of all CRCs core TFs across each cancer can be found in **Table 1**. We count the number of core TFs in each cancer to facilitate users to browse and search for core TFs according to each cancer.

**Table 1:** Data summary in the Cancer CRC.

Cancer types	Number of H3K27ac samples	Average number of core TFs per sample	Number of CRCs	Number of core TFs
BLCA	1	27	87	27
BRCA	15	11.07	7707	166
CESC	2	22	306	44
COAD	16	9.88	68535	158
ESCA	2	25.5	1098	51
GBM	4	21	350	84
KIRC	3	20.67	5483	62
LIHC	1	34	287	34
LUAD	4	17	547	68
PCPG	23	5.57	5985	128
PRAD	4	19.75	791	79
SKCM	5	17.2	2701	86
STAD	15	4.33	231	65

TF, transcription factor; CRC, core transcriptional regulatory circuitries.

### Other Annotation Information in the Cancer CRC

#### TF Degree

The total in-degree of a TF was defined as the sum of the TFs binding to their regulatory elements, while the out-degree of a TF was defined as the sum of the TFs that were regulated by it in the network. The total degree was calculated by summing the total number of edges adjacent to the given node (**Table S4**).

#### TF Frequency

In a sample, the frequency of a TF was defined as the number of all CRCs in the sample divided by the number of CRCs that contain that TF.

#### CRCs Score

“Clique score” was defined as the average TF frequency across all cliques of a sample.

#### TF Expression

Cancer CRC provides TF expression information in different samples. We downloaded mRNA expression profiles with FPKM values from TCGA (https://tcga-data.nci.nih.gov/tcga), GTEx, CCLE and ENCODE databases. The expression panel provides users with the differential expression of TFs in different samples.

#### Mutation Information

Several studies have shown that disease-associated variants are significantly enriched in TFs. Somatic mutations within TFs may affect the regulation of target genes and the expression of downstream genes (38–40). Thus, we collected somatic mutations in TFs from OncoBase (41). Furthermore, genetic mutations located in regulatory regions may provide clues that the mutation results in the dysregulation of gene expression (42, 43). We also annotated mutation information in TF regulatory regions, such as common SNPs, risk-associated SNPs, eQTLs, SAS, EAS, EUR, AMR and AFR.

#### GO Term Information

The GO database was used to predict the potential functions of TFs (44). We annotated biological terms to TFs and provided associated information for core TFs including GO_term_type and GO_term_name acquired from the GO database.

#### Disease Information

The Comparative Toxicogenomics Database (45) and PsyGeNE (46) pipelines were integrated into the Cancer CRC to evaluate the correlations between TFs and various diseases.

#### Pathway Information

Pathways associated with TFs were acquired from ComPAT (http://bio.licpathway.net:8018/msg/ComPAT/index.do). For a core TF, we provided related pathways as well as the pathway name, pathway source, gene number and edge number of the current pathway. When selecting the pathway ID, detailed information including comprehensive annotations and interactive visualizations of the current pathway are shown.

#### Survival Analysis

The survival analysis module allows users to select a TF or multiple TFs on the detail page for survival analysis, which is implemented by the built-in “gepia2” python package (47). Furthermore, there are several clinical factors, such as age, sex, stage, etc. to help users observe the impact of these factors on prognosis.

## Results

### Construction and Functionalities of the Cancer CRC

**Figure 1:**
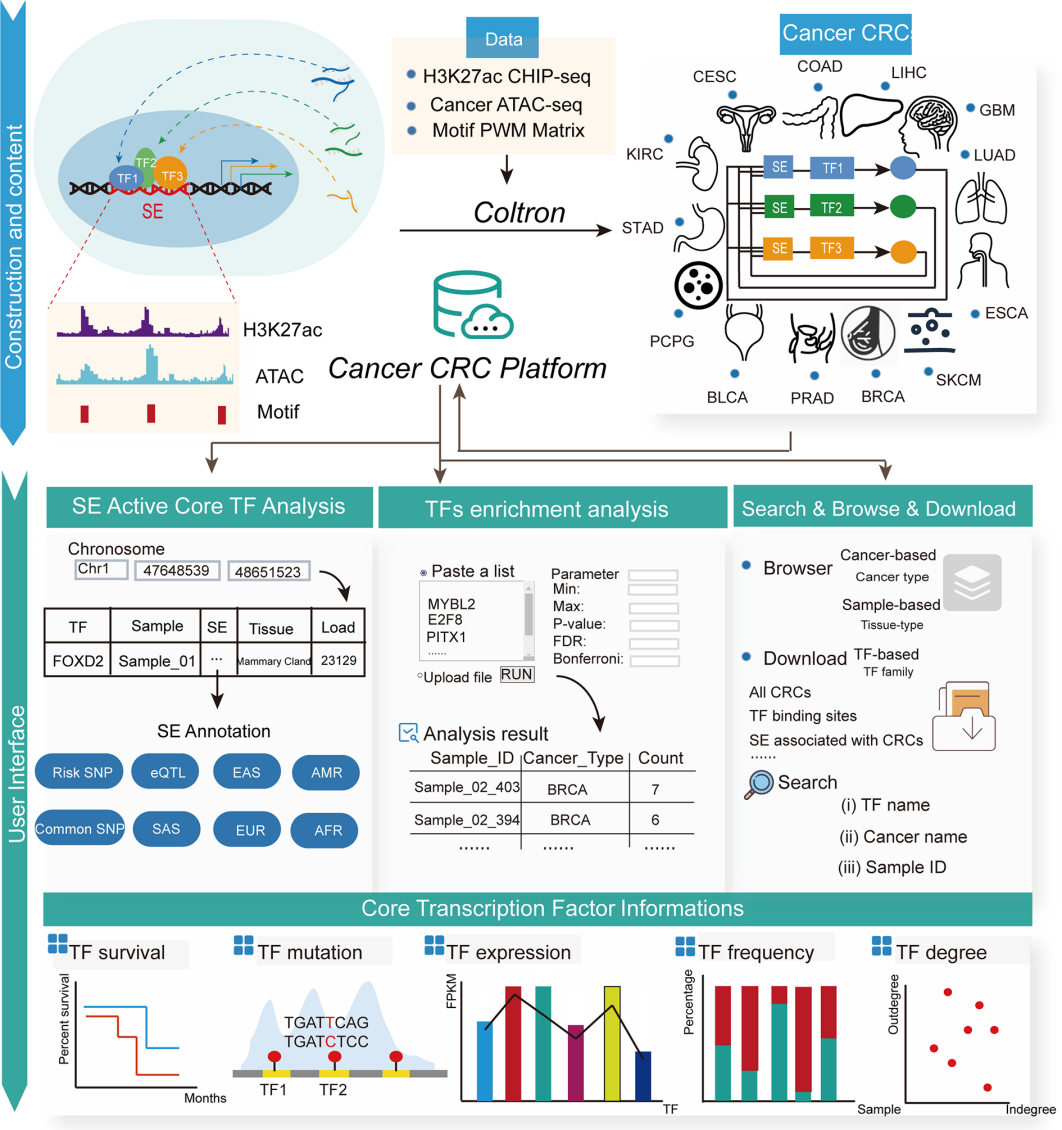
The user interface of the Cancer CRC platform. The Cancer CRC provides CRCs and core TFs for 13 types of cancer, including bladder urothelial carcinoma, breast cancer, colon adenocarcinoma and esophageal carcinoma, among others. The Cancer CRC supports multiple functions, including search, download, browse and enrichment analysis. Additionally, for core TFs, there is extensive annotation information, such as TF survival, mutations, expression, frequency and degree in CRCs.

The construction of the Cancer CRC platform was based on the comprehensive identification and analysis of CRCs from various cancer types. The content and construction pipeline of the platform are presented in **Figure 1**. To facilitate the broad usage of the platform, we developed a user-friendly, interactive open-access web portal for querying and visualizing detailed information on CRCs and core TFs. Each chart or table in the Cancer CRC is available for download. Briefly, this resource contains extensive annotation information on CRCs and core TFs. The Cancer CRC also provides two practical TF analysis tools. The current version of the Cancer CRC platform was organized with MySQL 5.7.17 (http://www.mysql.com/) and runs on a Linux-based Aliyun Web server. The website was developed based on PHP5.4.45.0 (https://www.php.net/), with CSS3 and HTML5 frameworks. We designed and built the web interface using Bootstrap v3.3.7 (https://v3.bootcss.com/) and JQuery v2.1.1 (http://jquery.com).

### Online Analysis Tools

Cancer CRC provides two tools to perform related analyses on core TFs. In the first tool, “Super enhancer active core TF analysis”, users can obtain relevant TF regulatory information by inputting a genomic region. Then, SE regions overlapping with the input region will be returned, as well as core TFs regulated by these SE regions and linked sample information. The core TFs related to CRC information can also be obtained by clicking on a specific TF. Furthermore, for the SE regions, we provide detailed annotation information including common single nucleotide polymorphisms (SNPs), risk-associated SNPs and expression quantitative trait loci (eQTLs), which may affect the binding of TFs to SEs. Second, in the “TF enrichment analysis” tool, Cancer CRC annotates TFs submitted by users with reference cancer core TF sets. We calculated the statistical significance of the enrichment analysis using the hypergeometric test (48). The corresponding enrichment results are shown in the form of a bubble diagram, bar graphs and a Venn diagram. Furthermore, Cancer CRC provides further information on these annotated TFs.

### Personalized Genome Browser and Data Visualization

The query on the search page is limited to TFs. To better display multidimensional TF information across the entire genome, we developed a personalized genome browser using JBrowse (49) to view detailed transcriptional regulatory information with several tracks. Users can select multiple tracks to interactively display peaks at a specific genomic location and see the proximity of specific regions to nearby genes, genomic segments, SNPs, common SNPs, risk-associated SNPs, enhancers and conserved TF binding sites. For each genomic region in our platform, there is a hyperlink to the UCSC Genome Browser webserver and the built-in JBrowse viewer.

### An Application of Cancer CRC to Explore the CRCs in Esophageal Cancer

**Figure 2:**
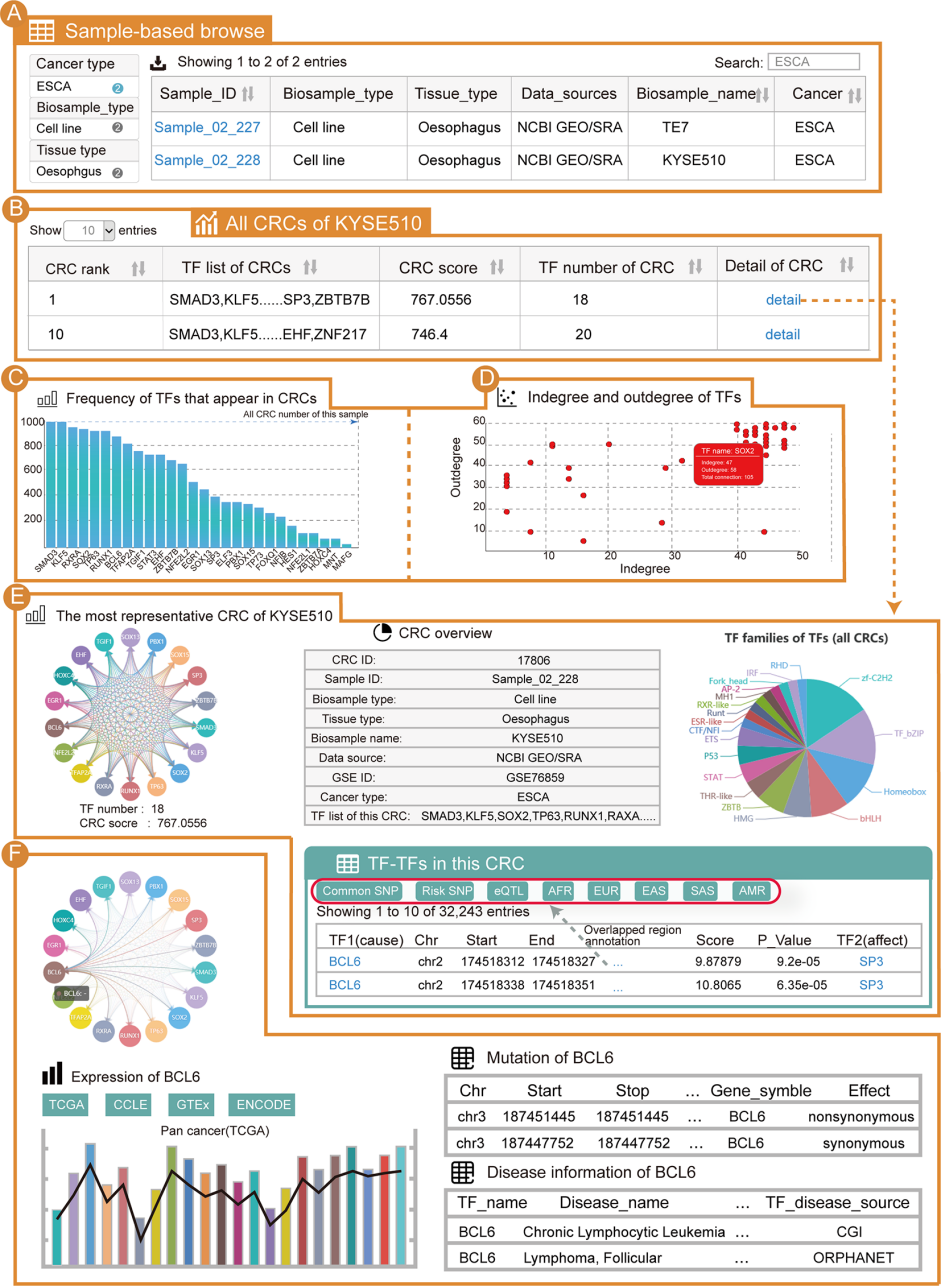
Use of Cancer CRC for esophageal carcinoma (ESCA) analysis. **(A)** Detailed sample information for ESCA. **(B)** All CRCs from the KYSE510 cell line (sample ID: Sample_02_228). **(C)** Frequency of TFs that are in the CRCs of Sample_02_228. **(D)** Scatter graph of TF degree of the CRCs in Sample_02_228. **(E)** Details of the most representative CRC in Sample_02_228. **(F)** Details of the TF *BCL6* in these CRCs.

From the “Browse” page, users can apply three strategies to search for samples of interest. To find esophageal cancer CRCs, users can select the “Cancer type” as “ESCA” (option 1); enter “ESCA” in the “Search” tab (option 2); or adjust the number of entries per page and look up their cancer of interest in the alphanumerical table (option 3). From the results, we can see that there are two samples of esophageal cancer (**Figure 2A**). After clicking on “Sample_02_228” and the KYSE510 cell line, the page will be redirected to display the sample details. For the current sample, all CRCs in this sample will be shown (**Figure 2B**). This table contains information for each CRC in this sample, including the TF list, CRC score, number of TFs and a hyperlink to the CRC detail page. For the core TFs in the current sample, one important piece of information is the frequency of the core TFs present in the CRC of that sample (**Figure 2C**). We found that *KLF5, SOX2* and *TP63* all have a high frequency in this sample’s CRCs. Additionally, all of the core TFs have a well-established function in esophageal cancer (50–52). Another significant piece of information on the core TFs is the degree (**Figure 2D**). Notably, *KLF5, SOX2* and *TP63* were also highly connected in the KYSE510 cell line. We found that TFs with higher frequencies also tended to have higher degree, and these two properties of core TFs could help select TFs that are important in cancers. In addition, information on the sample details also included: (i) a sample overview (sample basic information, number of CRCs in this sample and all core TFs of this sample); (ii) the most representative CRC of this sample; (iii) the “Expression” panel shows the differential expression of core TFs across a large panel of human cell lines, normal tissues and cancers.

Taking the most representative CRC of esophageal cancer Sample_02_228 as an example, the CRC details page will be redirected to display a visual and interactive CRC model along with the exportable tabular information on individual core TFs. Cancer CRC provides regulatory information on TFs in one CRC. The genetic and epigenetic annotations for binding regions of TFs, including common SNPs, risk-associated SNPs, eQTLs, and so on, are provided (**Figure 2E**). Furthermore, conjoint survival analysis and co-expression of TFs in each CRC are provided on the CRC detail pages. When clicking on a TF (e.g., *BCL6*), the mutation, disease and expression information of that TF are displayed (**Figure 2F**). Moreover, information on individual core TFs, their corresponding SE regions, the frequencies of core TFs in all of the CRCs of the sample, the distribution of core TFs among different CRCs (the distribution of a TF in samples most representative CRC and the distribution of a TF in all CRCs), survival analysis results, TF associated pathway information and GO term information are provided on the detail page. It is worth mentioning that if users want to search for esophageal cancer related core TFs through the search page, our platform can rank all the core TFs in esophageal cancer (section “TF in CRCs of BRCA”) on the return page to help users find potentially important TFs in esophageal cancer.

### A Case Study Using Differential Expressed Breast Cancer TFs

**Figure 3:**
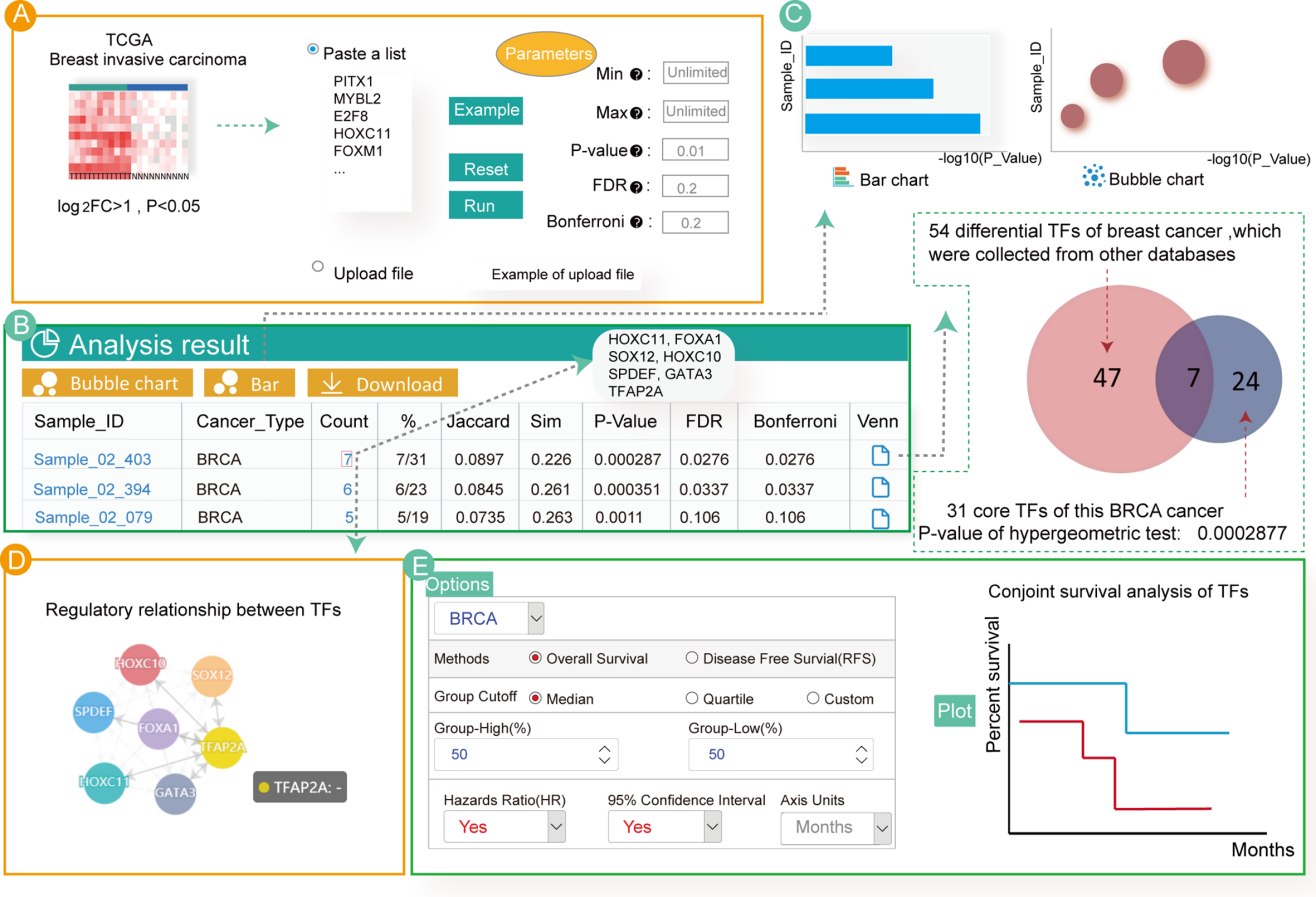
Enrichment analyses associated with 54 up-regulated TFs in breast cancer. **(A)** Input and parameter selection for the enrichment analysis. **(B)** Results table for the enrichment analyses. **(C)** Visualization of the enrichment analysis results. **(D)** Regulatory relationship among the annotated TFs. **(E)** Conjoint survival analysis for the annotated TFs.

Numerous studies have focused on enrichment analysis of differentially expressed TFs to explore their potential functions. Therefore, by analyzing differentially expressed TFs in breast cancer, we aimed to find key TFs affecting its occurrence and development. First, we used the “limma” R package (53) to calculate differentially expressed genes in breast cancer samples from TCGA. Then, we obtained the differentially expressed TFs (log_2_FC > 1, *P* < 0.05) (**Table S5**) as input for Cancer CRC. Next, we set the parameters of the hypergeometric test at *P* = 0.01 and clicked the “RUN” button to start the enrichment analysis (**Figure 3A**).

The analytical results showed that these TFs were only significantly enriched in three invasive breast cancer samples (**Figure 3B**). Users can click on the hyperlink to view the sample details and TFs annotated to the sample. In total, seven TFs (*HOXC11, FOXA1, SPDEF, SOX12, HOXC10, GATA3* and *TFAP2A*) were annotated to the breast cancer samples. Indeed, most of these TFs have previously been associated with breast cancer. For example, *HOXC11* was identified to be an indicator of poor response to hormonal therapy in breast cancer cases (54). *FOXA1, GATA3* and *SPDEF* are key drivers of estrogen receptor-positive (ER+) breast cancer risk as well as cancer progression (55, 56). In breast cancer cells, overexpression of *SOX12* partially improved the inhibitory effect on cell proliferation, migration and invasion. Additionally, a study demonstrated that cases with high *HOXC10* expression showed shorter recurrence-free and overall survival in patients with breast cancer undergoing chemotherapy (57, 58). However, one of the TFs annotated in our study, *TFAP2A*, has not been extensively studied in breast cancer, and may be a new critical TF associated with the disease. The bubble diagram, bar graphs and Venn diagram of the enrichment analysis result are also provided (**Figure 3C**). Users can download these results by clicking on the top button.

Although our analyses showed that these differentially expressed TFs are highly related to breast cancer, further exploration of the biological mechanisms leading to cancer remains essential. Therefore, we examined the regulatory relationship of the enriched TFs and found that all the identified TFs regulated each other (**Figure 3D**). Furthermore, customized survival analysis could guide research on prognosis for these TFs (**Figure 3E**).

Taken together, we provided a specific case study of enrichment analysis for differentially expressed TFs in breast cancer using the Cancer CRC resource. Most of the annotated TFs had previously been shown to be directly related to breast cancer. These results showed the value and significance of our platform for research of TFs in cancer.

### Landscape of Core TFs Across Cancer Types

**Figure 4:**
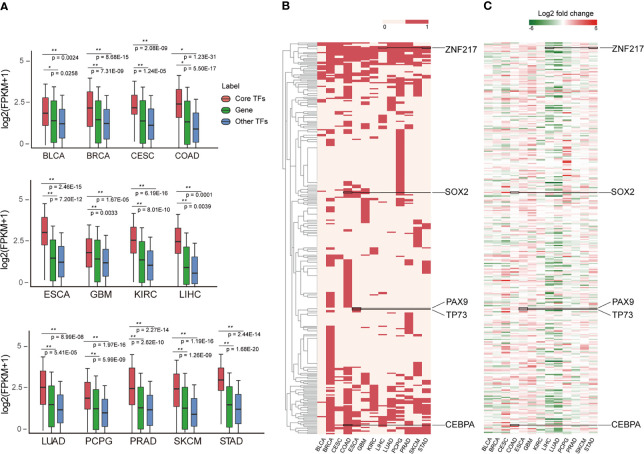
Landscape of core TFs in 13 cancers. **(A)** Box plots show the expression level on the log2 scale (FPKM+1) of core TFs, common genes and other TFs in 13 cancer types. P-values quantifying the difference between two sets were calculated using the Wilcoxon test. **(B)** Clustering of core TFs in different cancer types. Red represents the presence of TFs in the CRC of the cancer type. **(C)** Clustering of differently expressed core TFs in cancer types. The values were calculated by log_2_ (fold change) between control and cancer samples.

Investigating the expression patterns and distribution of all core TFs across cancer types can reveal conserved and cancer-specific TFs, which will help reveal their key roles in the genesis and progression of tumors. By analyzing mRNA expression data, we found that the expression of core TFs across cancers was significantly higher than non-TF mRNA and other TFs (**Figure 4A**), suggesting that core TFs were closely associated with cancer developmental processes (17). Next, we examined the frequency of the core TFs in each cancer type, and we found a lot of universal and specific core TFs in various cancers (**Figure 4B**). On one hand, one group of TFs was specific for certain cancers. Cancer-specific TFs play a key role in the occurrence and development of different cancers. For example, *PAX9* and *TP73* appeared specifically in esophagus cancer (**Figure 4B**). Studies have shown that *PAX9* regulated squamous cell differentiation in the epithelium, and progressive loss of *PAX9* expression correlated with increasing malignancy of the esophagus (59, 60). However, the specific TF *MITF* of skin cutaneous melanoma (SKCM), which is a marker of melanoma cell differentiation and regulates a variety of melanocyte differentiation genes. It has been reported that the down-regulation of *MITF* is related to the acquisition of melanoma stem cells, and *MITF* is involved in the metastatic growth of melanoma after diffusion. Besides, the SKCM specific TFs *GLI3*, *MEF2C*, and *MITF* are closely related to melanocyte differentiation, pigment cell differentiation and pigmentation pathway (61–63). For esophageal cancer, as shown in **Figure 4C**, *PAX9* was expressed at a low level in esophageal cancer. For *TP73*, a study showed that a SNP (rs2273953) in *TP73* in esophageal cancer patients impacted disease-free survival (64). Overall, these TFs play important roles in the occurrence and development of esophageal cancer. On the other hand, some TFs were prevalent across digestive tract cancers, such as *SOX2, CEBPA* and *ZNF217*. Among them, *SOX2* expression may serve as a novel prognostic factor for patients with digestive tract cancers. Overexpression of *SOX2* is correlated with vascular invasion, differentiation and poor prognosis of patients with digestive tract cancers (65). Additionally, the overexpression of *SOX2* in digestive tract cancer is evident in **Figure 4C**. Many studies have confirmed that *CEPBA* was a master regulator of hepatic function, and *CEPBA* expression was suppressed in many liver diseases including hepatocellular carcinoma (66). Additionally, *CEBPA* has been identified as a gene with high frequency mutations in hepatocellular adenocarcinoma of the stomach (a type of gastric cancer) (67). Finally, *ZNF217* is overexpressed in colorectal carcinoma tissues and is associated with tumor malignant clinicopathological features, which may promote colorectal carcinoma progression by inducing cell migration and invasion (68, 69). In addition, *ZNF217* expression may be a novel prognostic biomarker in gastric cancer (70). The expression of these TFs in different cancers are provided in **Figure 4C**, and the expression levels of different TFs in corresponding cancers were consistent with previous studies (59, 60, 65, 66, 68, 69). Next, in **Figure S2A**, we demonstrate the distribution of cancer-specific core TFs (core TFs that are present only in one cancer type) in cancers. Together with **Figure 4B**, we describe the distribution of all core TFs in different cancers and discovered the specificity of core TFs across different cancer types. Furthermore, we found that the expression of these specific core TFs also showed specificity in different cancer types (**Figure S2**).

## Discussion

The general mechanisms underlying gene transcription are well understood (71, 72), but the regulatory patterns of gene expression programs controlled by a small number of TFs are unknown in most cells. Identification of CRCs in tumors can reveal clues relating to cellular origin and gene regulatory drivers of the oncogenic state, which may offer novel strategies to fight against malignancy. To accurately identify CRCs in different cancer types, the current release of the Cancer CRC platform integrates publicly available cancer ChIP-seq and ATAC-seq datasets.

The Cancer CRC platform provides a user-friendly interface to search, browse, analyze and visualize information on cancer CRCs. Cancer CRC has abundant annotations, visual charts and useful analysis tools. Cancer CRC provides: (i) comprehensive information on core TFs, including the topological properties of TFs (in-degree and out-degree), expression values of TFs, frequencies of core TFs in the CRC from each sample, and the SEs, mutations, disease information, pathways, GO functions and survival information related to these TFs, all of which have user-friendly displays with interactive tables; (ii) the ability to search related CRCs based on cancer name, H3k27ac Sample ID or TF names; (iii) the ability to browse each sample to obtain detailed information; (iv) online analysis tools for “Super enhancer active core TFs analysis” and “TF enrichment analysis”; (v) a customized genome browser for user-friendly visualization of genomic context information; and (vi) curated data for each sample along with visual charts that can be downloaded from the platform.

In our pipeline, in order to improve the accuracy of cancer CRCs, we integrate tissues/cell lines H3K27ac ChIP-seq data and matched cancer ATAC-seq data. However, TCGA project only published ATAC-seq data for 23 cancers and researchers usually selectively implemented H3K27ac ChIP-seq experiments in some cancer cell lines, which led to the small sample size of our study. Fortunately, with the rapid development of high-throughput sequencing technology, more cancer accessible chromatin data will be available, and cell line/tissie-specific H3K27ac data will be greatly increased, which enable us to obtain more CRCs and core TFs for various cancers and expand our platform resources in future. On this basis, we will also develop a comprehensive algorithm that integrate multiple properties of core TFs to help researchers better identify potentially TFs in cancers.

Because our platform provides cell line/tissue-specific TFs, it can be used to explore the target genes regulated by core TFs in a CRC. For a specific sample of a cancer, we can find all the target genes regulated by all the core TFs in CRCs from SEdb (29), so as to obtain the common target genes regulated by core TFs. Next, for a CRC, the list of target genes regulated by all core TFs of this CRC can be get. Then the number of target genes regulated by each core TF can be calculated, and we can find out the genes more jointly regulated by a CRC, which are more likely to be tissue-specific target genes. In future updated version of Cancer CRC, we will also consider adding such data for easy use by researchers. Furthermore, we will integrate TF ChIP-seq data to predict target genes and add them to our platform in the future.

## Conclusions

Our motivation to establish this platform was prompted by the need to understand both cell/tissue type-specific TFs and CRCs in different cancer types. Systematic identification of active regulatory elements in cells has led to several key observations (26). By constructing specific TF regulatory networks using specific cancer enhancer profiles and chromatin accessibility regions, we identified critical TF nodes and CRCs. In addition, we systematically analyzed the core TFs across cancer types, providing a valuable reference for cancer research. We anticipate that these analyses will provide a framework for describing the state of primary tumor and will be useful in identifying TFs as potential therapeutic targets.

## Data Availability Statement

The original contributions presented in the study are included in the article/**Supplementary Material**. Further inquiries can be directed to the corresponding authors.

## Author Contributions

LW, JZ, and CL designed the research. LW, CF, MX, YG, XZ, FQ, YuZ, YiZ, YaZ, DS, JL, and QW performed the research. LW, JC, YJ, and XW performed the analyses and built the web interface. LW, CS, and JZ wrote the manuscript. All authors contributed to the article and approved the submitted version.

## Funding

This work was supported by National Natural Science Foundation of China [62171166]; Natural Science Foundation for Distinguished Young Scholars of Heilongjiang Province of China [JQ2020C004]; Research Foundation of the First Affiliated Hosptial of University of South China for Advanced Talents; Open Project of Centre of Diabetic Systems Medicine, Guangxi Key Laboratory of Excellence, Guilin Medical University; National Natural Science Foundation of China [62001145, 81572341]; Wu liande Youth Training Fund of Harbin Medical University [JFWLD202001]; Innovative Young Talents Training Scheme for tertiary institutions of Heilongjiang Province [UNPYSCT-2020164]; Health Council Research Program of Heilongjiang Province [2019-075].

## Conflict of Interest

The authors declare that the research was conducted in the absence of any commercial or financial relationships that could be construed as a potential conflict of interest.

## Publisher’s Note

All claims expressed in this article are solely those of the authors and do not necessarily represent those of their affiliated organizations, or those of the publisher, the editors and the reviewers. Any product that may be evaluated in this article, or claim that may be made by its manufacturer, is not guaranteed or endorsed by the publisher.

## References

[1] Mitchell PJ Tjian R. Transcriptional Regulation in Mammalian Cells by Sequence-Specific DNA Binding Proteins. Science (1989) 245:371–8. doi: 10.1126/science.2667136 2667136

[2] Graf T Enver T. Forcing Cells to Change Lineages. Nature (2009) 462:587–94. doi: 10.1038/nature08533 19956253

[3] Takahashi K Tanabe K Ohnuki M Narita M Ichisaka T Tomoda K. Induction of Pluripotent Stem Cells From Adult Human Fibroblasts by Defined Factors. Cell (2007) 131:861–72. doi: 10.1016/j.cell.2007.11.019 18035408

[4] Lee TI Young RA. Transcriptional Regulation and its Misregulation in Disease. Cell (2013) 152:1237–51. doi: 10.1016/j.cell.2013.02.014 PMC3640494 23498934

[5] Whyte WA Orlando DA Hnisz D Abraham BJ Lin CY Kagey MH. Master Transcription Factors and Mediator Establish Super-Enhancers at Key Cell Identity Genes. Cell (2013) 153:307–19. doi: 10.1016/j.cell.2013.03.035 PMC3653129 23582322

[6] Neph S Stergachis AB Reynolds A Sandstrom R Borenstein E Stamatoyannopoulos JA. Circuitry and Dynamics of Human Transcription Factor Regulatory Networks. Cell (2012) 150:1274–86. doi: 10.1016/j.cell.2012.04.040 PMC3679407 22959076

[7] Durbin AD Zimmerman MW Dharia NV Abraham BJ Iniguez AB Weichert-Leahey N. Selective Gene Dependencies in MYCN-Amplified Neuroblastoma Include the Core Transcriptional Regulatory Circuitry. Nat Genet (2018) 50:1240–6. doi: 10.1038/s41588-018-0191-z PMC6386470 30127528

[8] Ran L Chen Y Sher J Wong EWP Murphy D Zhang JQ. FOXF1 Defines the Core-Regulatory Circuitry in Gastrointestinal Stromal Tumor. Cancer Discov (2018) 8:234–51. doi: 10.1158/2159-8290.CD-17-0468 PMC5809271 29162563

[9] Boyer LA Lee TI Cole MF Johnstone SE Levine SS Zucker JP. Core Transcriptional Regulatory Circuitry in Human Embryonic Stem Cells. Cell (2005) 122:947–56. doi: 10.1016/j.cell.2005.08.020 PMC3006442 16153702

[10] Wang Z Oron E Nelson B Razis S Ivanova N. Distinct Lineage Specification Roles for NANOG, OCT4, and SOX2 in Human Embryonic Stem Cells. Cell Stem Cell (2012) 10:440–54. doi: 10.1016/j.stem.2012.02.016 22482508

[11] Kim J Chu J Shen X Wang J Orkin SH. An Extended Transcriptional Network for Pluripotency of Embryonic Stem Cells. Cell (2008) 132:1049–61. doi: 10.1016/j.cell.2008.02.039 PMC3837340 18358816

[12] Marson A Levine SS Cole MF Frampton GM Brambrink T Johnstone S. Connecting Microrna Genes to the Core Transcriptional Regulatory Circuitry of Embryonic Stem Cells. Cell (2008) 134:521–33. doi: 10.1016/j.cell.2008.07.020 PMC2586071 18692474

[13] Chew JL Loh YH Zhang W Chen X Tam WL Yeap LS. Reciprocal Transcriptional Regulation of Pou5f1 and Sox2 *via* the Oct4/Sox2 Complex in Embryonic Stem Cells. Mol Cell Biol (2005) 25:6031–46. doi: 10.1128/MCB.25.14.6031-6046.2005 PMC1168830 15988017

[14] Chen L Huang M Plummer J Pan J Jiang YY Yang Q. Master Transcription Factors Form Interconnected Circuitry and Orchestrate Transcriptional Networks in Oesophageal Adenocarcinoma. Gut (2020) 69:630–40. doi: 10.1136/gutjnl-2019-318325 PMC8108390 31409603

[15] Jiang YY Jiang Y Li CQ Zhang Y Dakle P Kaur H. TP63, SOX2, and KLF5 Establish a Core Regulatory Circuitry That Controls Epigenetic and Transcription Patterns in Esophageal Squamous Cell Carcinoma Cell Lines. Gastroenterology (2020) 159:1311–27.e19. doi: 1 10.1053/j.gastro.2020.06.050 32619460

[16] Decaesteker B Denecker G Van Neste C Dolman EM Van Loocke W Gartlgruber M. TBX2 Is a Neuroblastoma Core Regulatory Circuitry Component Enhancing MYCN/FOXM1 Reactivation of DREAM Targets. Nat Commun (2018) 9:4866. doi: 10.1038/s41467-018-06699-9 30451831 PMC6242972

[17] Saint-Andre V Federation AJ Lin CY Abraham BJ Reddy J Lee TI. Models of Human Core Transcriptional Regulatory Circuitries. Genome Res (2016) 26:385–96. doi: 10.1101/gr.197590.115 PMC4772020 26843070

[18] Chapuy B McKeown MR Lin CY Monti S Roemer MG Qi J. Discovery and Characterization of Super-Enhancer-Associated Dependencies in Diffuse Large B Cell Lymphoma. Cancer Cell (2013) 24:777–90. doi: 10.1016/j.ccr.2013.11.003 PMC4018722 24332044

[19] Christensen CL Kwiatkowski N Abraham BJ Carretero J Al-Shahrour F Zhang T. Targeting Transcriptional Addictions in Small Cell Lung Cancer With a Covalent CDK7 Inhibitor. Cancer Cell (2014) 26:909–22. doi: 10.1016/j.ccell.2014.10.019 PMC4261156 25490451

[20] Gryder BE Yohe ME Chou HC Zhang X Marques J Wachtel M. PAX3-FOXO1 Establishes Myogenic Super Enhancers and Confers BET Bromodomain Vulnerability. Cancer Discov (2017) 7:884–99. doi: 10.1158/2159-8290.CD-16-1297 PMC7802885 28446439

[21] Hnisz D Abraham BJ Lee TI Lau A Saint-Andre V Sigova AA. Super-Enhancers in the Control of Cell Identity and Disease. Cell (2013) 155:934–47. doi: 10.1016/j.cell.2013.09.053 PMC3841062 24119843

[22] Loven J Hoke HA Lin CY Lau A Orlando DA Vakoc CR. Selective Inhibition of Tumor Oncogenes by Disruption of Super-Enhancers. Cell (2013) 153:320–34. doi: 10.1016/j.cell.2013.03.036 PMC3760967 23582323

[23] Huang M Chen Y Yang M Guo A Xu Y Xu L. Dbcorc: A Database of Core Transcriptional Regulatory Circuitries Modeled by H3k27ac Chip-Seq Signals. Nucleic Acids Res (2018) 46:D71–7. doi: 10.1093/nar/gkx796 PMC5753200 28977473

[24] Mei S Qin Q Wu Q Sun H Zheng R Zang C. Cistrome Data Browser: A Data Portal for Chip-Seq and Chromatin Accessibility Data in Human and Mouse. Nucleic Acids Res (2017) 45:D658–62. doi: 10.1093/nar/gkw983 PMC5210658 27789702

[25] Federation AJ Polaski DR Ott CJ Fan A Lin CY Bradner JE. Identification of Candidate Master Transcription Factors Within Enhancer-Centric Transcriptional Regulatory Networks. bioRxiv (2018). doi: 10.1101/345413

[26] Ott CJ Federation AJ Schwartz LS Kasar S Klitgaard JL Lenci R. Enhancer Architecture and Essential Core Regulatory Circuitry of Chronic Lymphocytic Leukemia. Cancer Cell (2018) 34:982–95.e7. doi: 10.1016/j.ccell.2018.11.001 30503705 PMC6298230

[27] Langmead B Trapnell C Pop M Salzberg SL. Ultrafast and Memory-Efficient Alignment of Short DNA Sequences to the Human Genome. Genome Biol (2009) 10:R25. doi: 10.1186/gb-2009-10-3-r25 19261174 PMC2690996

[28] Zhang Y Liu T Meyer CA Eeckhoute J Johnson DS Bernstein BE. Model-Based Analysis of Chip-Seq (MACS). Genome Biol (2008) 9:R137. doi: 10.1186/gb-2008-9-9-r137 18798982 PMC2592715

[29] Jiang Y Qian F Bai X Liu Y Wang Q Ai B. Sedb: A Comprehensive Human Super-Enhancer Database. Nucleic Acids Res (2019) 47:D235–43. doi: 10.1093/nar/gky1025 PMC6323980 30371817

[30] Qian FC Li XC Guo JC Zhao JM Li YY Tang ZD. Seanalysis: A Web Tool for Super-Enhancer Associated Regulatory Analysis. Nucleic Acids Res (2019) 47:W248–55. doi: 10.1093/nar/gkz302 PMC6602466 31028388

[31] Corces MR Granja JM Shams S Louie BH Seoane JA Zhou W. The Chromatin Accessibility Landscape of Primary Human Cancers. Science (2018) 362(6413):eaav1898. doi: 10.1126/science.aav1898 30361341 PMC6408149

[32] Karolchik D Barber GP Casper J Clawson H Cline MS Diekhans M. The UCSC Genome Browser Database: 2014 Update. Nucleic Acids Res (2014) 42:D764–70. doi: 10.1093/nar/gkt1168 PMC3964947 24270787

[33] Zhang H Zhu L Huang DS. Discmla: An Efficient Discriminative Motif Learning Algorithm Over High-Throughput Datasets. IEEE/ACM Trans Comput Biol Bioinform (2018) 15:1810–20. doi: 10.1109/TCBB.2016.2561930 27164602

[34] Zhang H Zhu L Huang DS. WSMD: Weakly-Supervised Motif Discovery in Transcription Factor Chip-Seq Data. Sci Rep (2017) 7:3217. doi: 10.1038/s41598-017-03554-7 28607381 PMC5468353

[35] Grant CE Bailey TL Noble WS. FIMO: Scanning for Occurrences of a Given Motif. Bioinformatics (2011) 27:1017–8. doi: 10.1093/bioinformatics/btr064 PMC3065696 21330290

[36] Mathelier A Zhao X Zhang AW Parcy F Worsley-Hunt R Arenillas DJ. JASPAR 2014: An Extensively Expanded and Updated Open-Access Database of Transcription Factor Binding Profiles. Nucleic Acids Res (2014) 42:D142–7. doi: 10.1093/nar/gkt997 PMC3965086 24194598

[37] Matys V Kel-Margoulis OV Fricke E Liebich I Land S Barre-Dirrie A. TRANSFAC and its Module Transcompel: Transcriptional Gene Regulation in Eukaryotes. Nucleic Acids Res (2006) 34:D108–10. doi: 10.1093/nar/gkj143 PMC1347505 16381825

[38] Miao Y Zou YX Gu DL Zhu HC Zhu HY Wang L. SF3B1 Mutation Predicts Unfavorable Treatment-Free Survival in Chinese Chronic Lymphocytic Leukemia Patients. Ann Transl Med (2019) 7:176. doi: 10.21037/atm.2019.03.63 31168457 PMC6526254

[39] Capasso M Lasorsa VA Cimmino F Avitabile M Cantalupo S Montella A. Transcription Factors Involved in Tumorigenesis are Over-Represented in Mutated Active DNA-Binding Sites in Neuroblastoma. Cancer Res (2020) 80:382–93. doi: 10.1158/0008-5472.CAN-19-2883 31784426

[40] Afzali B Gronholm J Vandrovcova J O’Brien C Sun HW Vanderleyden I. BACH2 Immunodeficiency Illustrates an Association Between Super-Enhancers and Haploinsufficiency. Nat Immunol (2017) 18:813–23. doi: 10.1038/ni.3753 PMC5593426 28530713

[41] Li X Shi L Wang Y Zhong J Zhao X Teng H. Oncobase: A Platform for Decoding Regulatory Somatic Mutations in Human Cancers. Nucleic Acids Res (2019) 47:D1044–55. doi: 10.1093/nar/gky1139 PMC6323961 30445567

[42] Agus ZS Goldberg M. Renal Mechanisms of the Natriuretic and Antiphosphaturic Effects of Triflocin–a New Diuretic. J Lab Clin Med (1970) 76:280–92. 5434007

[43] Koumakis E Giraud M Dieude P Cohignac V Cuomo G Airo P. Brief Report: Candidate Gene Study in Systemic Sclerosis Identifies a Rare and Functional Variant of the TNFAIP3 Locus as a Risk Factor for Polyautoimmunity. Arthritis Rheum (2012) 64:2746–52. doi: 10.1002/art.34490 22488580

[44] The Gene Ontology C. The Gene Ontology Resource: 20 Years and Still Going Strong. Nucleic Acids Res (2019) 47:D330–8. doi: 10.1093/nar/gky1055 PMC6323945 30395331

[45] Davis AP Grondin CJ Johnson RJ Sciaky D McMorran R Wiegers J. The Comparative Toxicogenomics Database: Update 2019. Nucleic Acids Res (2019) 47:D948–54. doi: 10.1093/nar/gky868 PMC6323936 30247620

[46] Gutierrez-Sacristan A Grosdidier S Valverde O Torrens M Bravo A Pinero J. Psygenet: A Knowledge Platform on Psychiatric Disorders and Their Genes. Bioinformatics (2015) 31:3075–7. doi: 10.1093/bioinformatics/btv301 PMC4565028 25964630

[47] Tang Z Li C Kang B Gao G Li C Zhang Z. GEPIA: A Web Server for Cancer and Normal Gene Expression Profiling and Interactive Analyses. Nucleic Acids Res (2017) 45:W98–102. doi: 10.1093/nar/gkx247 28407145 PMC5570223

[48] Yu G Wang LG Han Y He QY. Clusterprofiler: An R Package for Comparing Biological Themes Among Gene Clusters. Omics: J Integr Biol (2012) 16:284–7. doi: 10.1089/omi.2011.0118 PMC3339379 22455463

[49] Skinner ME Uzilov AV Stein LD Mungall CJ Holmes IH. Jbrowse: A Next-Generation Genome Browser. Genome Res (2009) 19:1630–8. doi: 10.1101/gr.094607.109 PMC2752129 19570905

[50] Ng CK Ma K Cheng Y Miyashita T Harmon JW Meltzer SJ. Kruppel-Like Factor 5 Promotes Sonic Hedgehog Signaling and Neoplasia in Barrett’s Esophagus and Esophageal Adenocarcinoma. Trans Oncol (2019) 12:1432–41. doi: 10.1016/j.tranon.2019.07.006 PMC6700477 31401336

[51] Ishida H Kasajima A Kamei T Miura T Oka N Yazdani S. SOX2 and Rb1 in Esophageal Small-Cell Carcinoma: Their Possible Involvement in Pathogenesis. Modern Pathol (2017) 30:660–71. doi: 10.1038/modpathol.2016.222 28106103

[52] Xie JJ Jiang YY Jiang Y Li CQ Lim MC An O. Super-Enhancer-Driven Long Non-Coding RNA LINC01503, Regulated by TP63, Is Over-Expressed and Oncogenic in Squamous Cell Carcinoma. Gastroenterology (2018) 154:2137–51.e1. doi: 10.1053/j.gastro.2018.02.018 29454790

[53] Ritchie ME Phipson B Wu D Hu Y Law CW Shi W. Limma Powers Differential Expression Analyses for RNA-Sequencing and Microarray Studies. Nucleic Acids Res (2015) 43:e47. doi: 10.1093/nar/gkv007 25605792 PMC4402510

[54] McIlroy M McCartan D Early S Gaora PO Pennington S Hill AD. Interaction of Developmental Transcription Factor HOXC11 With Steroid Receptor Coactivator SRC-1 Mediates Resistance to Endocrine Therapy in Breast Cancer [Corrected]. Cancer Res (2010) 70:1585–94. doi: 10.1158/0008-5472.CAN-09-3713 20145129

[55] Mehta RJ Jain RK Leung S Choo J Nielsen T Huntsman D. FOXA1 is an Independent Prognostic Marker for ER-Positive Breast Cancer. Breast Cancer Res Treat (2012) 131:881–90. doi: 10.1007/s10549-011-1482-6 21503684

[56] Campbell TM Castro MA Ponder BA Meyer KB. Identification of Post-Transcriptional Modulators of Breast Cancer Transcription Factor Activity Using Mindy. PLoS One (2016) 11:e0168770. doi: 10.1371/journal.pone.0168770 27997592 PMC5173250

[57] Du Y Shen L Zhang W Ding R Li Q Li S. Functional Analyses of Microrna-326 in Breast Cancer Development. Bioscience Rep (2019) 39(7):BSR20190787. doi: 10.1042/BSR20190787 PMC6663989 31311830

[58] Sadik H Korangath P Nguyen NK Gyorffy B Kumar R Hedayati M. HOXC10 Expression Supports the Development of Chemotherapy Resistance by Fine Tuning DNA Repair in Breast Cancer Cells. Cancer Res (2016) 76:4443–56. doi: 10.1158/0008-5472.CAN-16-0774 PMC4970943 27302171

[59] Xiong Z Ren S Chen H Liu Y Huang C Zhang YL. PAX9 Regulates Squamous Cell Differentiation and Carcinogenesis in the Oro-Oesophageal Epithelium. J Pathol (2018) 244:164–75. doi: 10.1002/path.4998 PMC5842438 29055049

[60] Gerber JK Richter T Kremmer E Adamski J Hofler H Balling R. Progressive Loss of PAX9 Expression Correlates With Increasing Malignancy of Dysplastic and Cancerous Epithelium of the Human Oesophagus. J Pathol (2002) 197:293–7. doi: 10.1002/path.1115 12115874

[61] Shen W Li Y Li B Zheng L Xie X Le J. Downregulation of KCTD12 Contributes to Melanoma Stemness by Modulating CD271. Cancer Biol Med (2019) 16:498–513. doi: 10.20892/j.issn.2095-3941.2019.0073 31565480 PMC6743620

[62] Simmons JL Pierce CJ Al-Ejeh F Boyle GM. MITF and BRN2 Contribute to Metastatic Growth After Dissemination of Melanoma. Sci Rep (2017) 7:10909. doi: 10.1038/s41598-017-11366-y 28883623 PMC5589904

[63] Perotti V Baldassari P Molla A Vegetti C Bersani I Maurichi A. Correction to: Nfatc2 is an Intrinsic Regulator of Melanoma Dedifferentiation. Oncogene (2019) 38:3763–4. doi: 10.1038/s41388-019-0679-8 PMC8075923 30692631

[64] Boonstra JJ van Marion R Tilanus HW Dinjens WN. Functional Polymorphisms Associated With Disease-Free Survival in Resected Carcinoma of the Esophagus. J Gastrointestinal Surgery (2011) 15:48–56. doi: 10.1007/s11605-010-1358-9 PMC3023032 20922573

[65] Du XM Wang LH Chen XW Li YX Li YC Cao YW. Prognostic Value of Sox2 Expression in Digestive Tract Cancers: A Meta-Analysis. J Huazhong Univ Sci Technology. Med Sci = Hua zhong ke ji da xue xue bao. Yi xue Ying wen ban = Huazhong keji daxue xuebao. Yixue Yingdewen ban (2016) 36:305–12. doi: 10.1007/s11596-016-1584-9 27376796

[66] Reebye V Huang KW Lin V Jarvis S Cutilas P Dorman S. Gene Activation of CEBPA Using Sarna: Preclinical Studies of the First in Human Sarna Drug Candidate for Liver Cancer. Oncogene (2018) 37:3216–28. doi: 10.1038/s41388-018-0126-2 PMC6013054 29511346

[67] Wang Y Sun L Li Z Gao J Ge S Zhang C. Hepatoid Adenocarcinoma of the Stomach: A Unique Subgroup With Distinct Clinicopathological and Molecular Features. Gastric Cancer: Off J Int Gastric Cancer Assoc Japanese Gastric Cancer Assoc (2019) 22:1183–92. doi: 10.1007/s10120-019-00965-5 PMC6811386 30989433

[68] Fang Z Xiong Y Zhang C Li J Liu L Li M. Coexistence of Copy Number Increases of ZNF217 and CYP24A1 in Colorectal Cancers in a Chinese Population. Oncol Lett (2010) 1:925–30. doi: 10.3892/ol_00000163 PMC3436466 22966406

[69] Zhang ZC Zheng LQ Pan LJ Guo JX Yang GS. ZNF217 is Overexpressed and Enhances Cell Migration and Invasion in Colorectal Carcinoma. Asian Pacific J Cancer Prevention: APJCP (2015) 16:2459–63. doi: 10.7314/APJCP.2015.16.6.2459 25824781

[70] Shida A Fujioka S Kurihara H Ishibashi Y Mitsumori N Omura N. Prognostic Significance of ZNF217 Expression in Gastric Carcinoma. Anticancer Res (2014) 34:4813–7. doi: 10.1245/s10434-014-3557-1 25202062

[71] Gifford CA Ziller MJ Gu H Trapnell C Donaghey J Tsankov A. Transcriptional and Epigenetic Dynamics During Specification of Human Embryonic Stem Cells. Cell (2013) 153:1149–63. doi: 10.1016/j.cell.2013.04.037 PMC3709577 23664763

[72] Ziller MJ Edri R Yaffe Y Donaghey J Pop R Mallard W. Dissecting Neural Differentiation Regulatory Networks Through Epigenetic Footprinting. Nature (2015) 518:355–9. doi: 10.1038/nature13990 PMC4336237 25533951

