# Supplementary figures and images for: Cancer CRC: A Comprehensive Cancer Core Transcriptional Regulatory Circuit Resource and Analysis Platform
Source: Front Oncol. 2021 Oct 12;11:761700. doi: 10.3389/fonc.2021.761700 (PMC8546348; doi:10.3389/fonc.2021.761700)

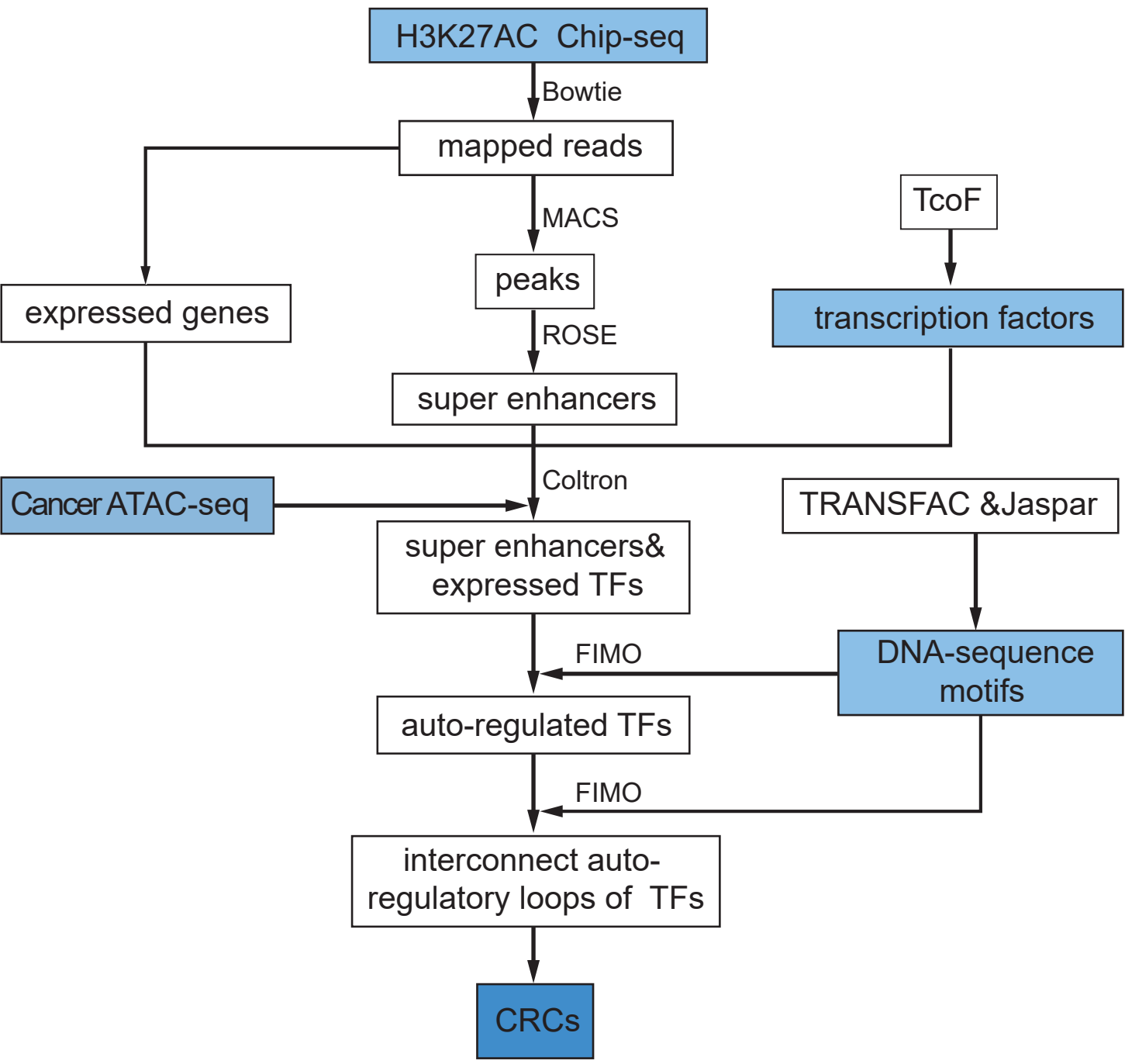

Supplement: Supplementary file 5 [file Image_1.pdf]

A

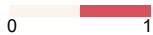

B

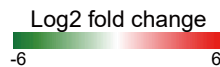

C

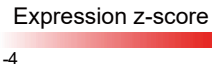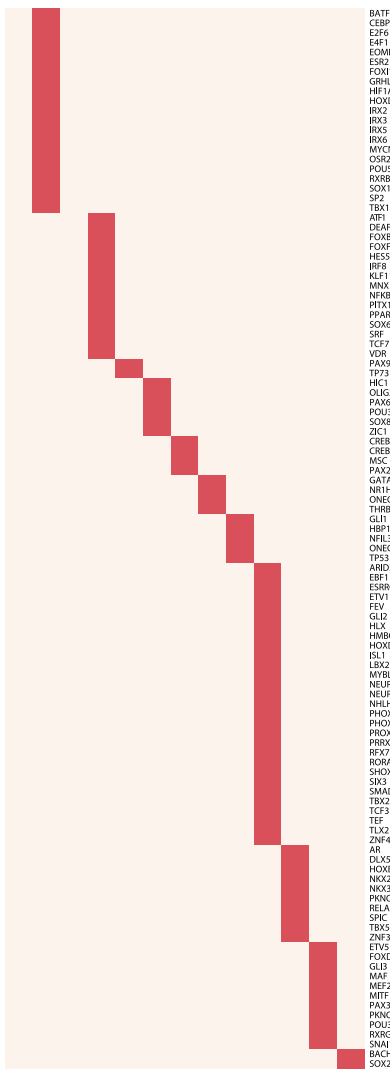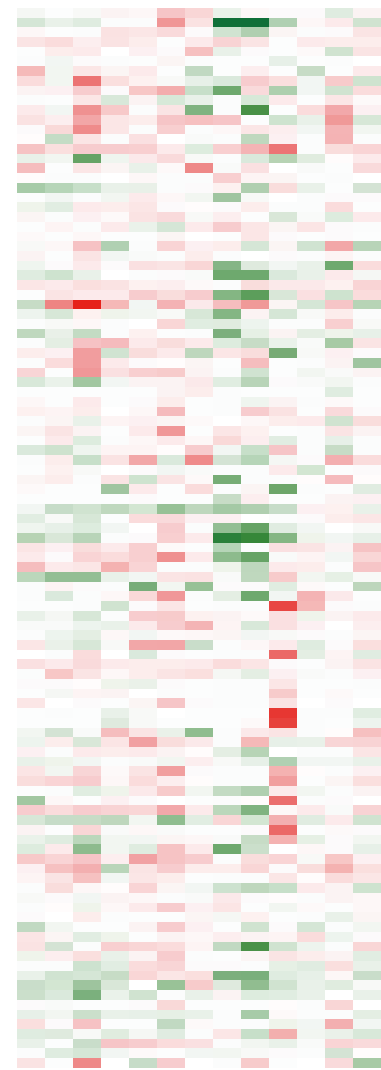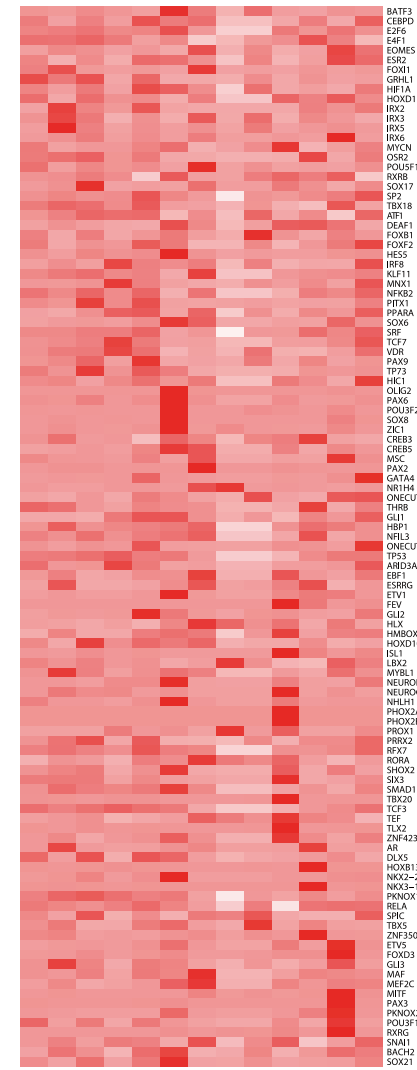

Supplement: Supplementary file 6 [file Image_2.pdf]
