# Supplementary material for: Cancer CRC: A Comprehensive Cancer Core Transcriptional Regulatory Circuit Resource and Analysis Platform
Source: Front Oncol. 2021 Oct 12;11:761700. doi: 10.3389/fonc.2021.761700 (PMC8546348; doi:10.3389/fonc.2021.761700)
Supplement: Supplementary file 7 [file Table_1.docx]

| **Data and functions** | **Description** | **Cancer CRC** | **dbCoRC** |
| --- | --- | --- | --- |
| Data | H3K27ac ChIP-seq | **√** | **√** |
|  | ATAC-seq | **√** |  |
| TF annotation | Expression | 5 sources | Only ENCODE |
|  | Mutation | **√** |  |
|  | Survival | **√** |  |
|  | Pathway | **√** |  |
|  | TF frequency in CRCs | **√** |  |
|  | Degree | **√** |  |
| Search CRCs | Search CRCs by cancers | **√** |  |
|  | Search CRCs by samples | **√** | **√** |
|  | Search CRCs by TFs | **√** | **√** |
| Genome browser | Super enhancer | **√** | **√** |
|  | Accessible chromatin | **√** |  |
|  | Conservation | **√** |  |
|  | Enhancer | **√** |  |
|  | SNP | **√** |  |
|  | TF Binding site | **√** |  |
| Analysis functions | Super-enhancer active TF analysis | **√** |  |
|  | TF enrichment analysis | **√** |  |

**Supplementary Table S1 Comparison of Cancer CRC with dbCoRC**
